# Multilayer network approaches to omics data integration in digital twins for cancer research

**DOI:** 10.3389/fsysb.2026.1776941

**Published:** 2026-06-04

**Authors:** Hugo Chenel, Malvina Marku, Tim James, Andrei Zinovyev, Vera Pancaldi

**Affiliations:** 1 Univ Toulouse, INSERM, CNRS, CRCT, Toulouse, France; 2 Equipe Labellisée Ligue Nationale Contre le Cancer, Paris, France; 3 Evotec, Toulouse, France; 4 Evotec, Abingdon, Oxfordshire, United Kingdom

**Keywords:** cancer systems biology, digital twins, multilayer networks, multi-omics integration, network medicine, precision oncology

## Abstract

How can we effectively integrate and represent heterogeneous multi-omics data to better understand complex biological systems and support the development of personalized digital models? The growing availability of high-dimensional omics data across different molecular scales presents both opportunities and challenges in biomedical research. Traditional approaches often treat each omics layer in isolation or rely on concatenation strategies that obscure the interactions between different regulatory layers. In this review, we discuss the multilayer network-based framework as an extensive representation of different omics data types, capturing the modularity, redundancy and cross-talk between layers, and providing a more faithful interpretable view of the biological system. We explore how this approach can be used as a basis for the construction of Digital Twins, computational replicas of individual biological systems capable of simulating disease progression and treatment outcomes. In contrast to existing multi-omics integration reviews), we emphasize the role of multilayer networks as a mechanistic and interpretable scaffold for Digital Twins development. We discuss key methodological considerations, benefits and potential applications of this approach, highlighting its promise for advancing both our understanding of biological complexity and our ability to design personalized interventions.

## Introduction

Precision oncology faces a major challenge: how to integrate heterogeneous multi-omics data into coherent and clinically actionable models of disease. The emergence of precision medicine warrants the development of tools that can exploit the vast and complex datasets generated by multi-omics approaches. These technologies, including (epi)genomics, transcriptomics, proteomics, metabolomics, metagenomics and other techniques that exhaustively characterize specific sets of biological entities, provide a comprehensive view of biological systems at multiple levels of regulation. Integrating these diverse types of datasets is essential to have an extensive and inclusive digital representation of patients and to identify novel biomarkers or therapeutic targets (Hasin et al., 2017), especially in cancer, which will be the focus of this review.

Digital Twins (DTs) have emerged as a transformative paradigm in healthcare, providing dynamic, *in silico* models of cells, organs, or entire organisms that are continuously updated with real-time data. These virtual counterparts enable predictive modeling and personalized interventions (Kamel Boulos and Zhang, 2021). DTs leverage advanced computational techniques to integrate and analyze large-scale multi-omics data to implement precision oncology. However, current DT approaches often rely on predictive or data-driven models that do not explicitly capture interactions across multiple biological scales, limiting their ability to provide mechanistic insights and to represent the multi-scale nature of cancer systems. From a systems biology point of view, an important type of DTs is based on network representations that span multiple scales and layers of biological information, often realized through multilayer networks. Multilayer networks (Kivela et al., 2014) have gained popularity for their ability to model complex systems where entities interact across multiple layers or dimensions. Early applications of multiplex biological networks demonstrated the value of propagating information across interconnected molecular layers for improved disease module identification (Valdeolivas et al., 2019; Baptista et al., 2024). In the context of multi-omics data integration, these multilayer networks can represent different types of biological information as interconnected layers, to capture the relationships and interactions between genes, proteins or other molecular entities (Lee et al., 2020; Trimbour et al., 2024), enabling the identification of crucial regulatory mechanisms and pathways that may remain elusive in single-layer network analyses, or using simple aggregation of these networks (De Domenico, 2023). Multilayer networks can be thought of as a mathematical foundation of knowledge graphs.

While the ongoing research in integrating multilayer networks in DTs shows promising outcomes (Vallée, 2026), several conceptual and practical challenges in their implementation for personalized medicine persist, including the intrinsic heterogeneous nature of multi-omics datasets, requiring elaborated algorithms (and potentially substantial computational resources). Ensuring data quality or consistency remains a significant obstacle. Finally, the development of reliable DTs requires robust validation methods to ensure that their predictive capabilities align with real-world outcomes (Hernandez-Boussard et al., 2021).

Despite these advances, there is still a lack of integrative frameworks that simultaneously preserve the structure of individual omics layers, model cross-layer interactions, and also support mechanistic interpretation required for DTs applications. In this review, we address both the importance and the challenges of integrating heterogeneous multi-omics datasets by adopting a multilayer network representation, which enables the modeling of complex biological interactions across molecular layers. This approach preserves the intrinsic structure of each omics layer while allowing modeling of inter-layer relationships, thus offering a more comprehensive and interpretable view of biological systems. We further discuss how this multilayer representation provides a foundational framework for the development of DTs in medicine that can simulate individual-specific disease progression and therapeutic responses.

## Multi-omics data integration approaches

**FIGURE 1:**
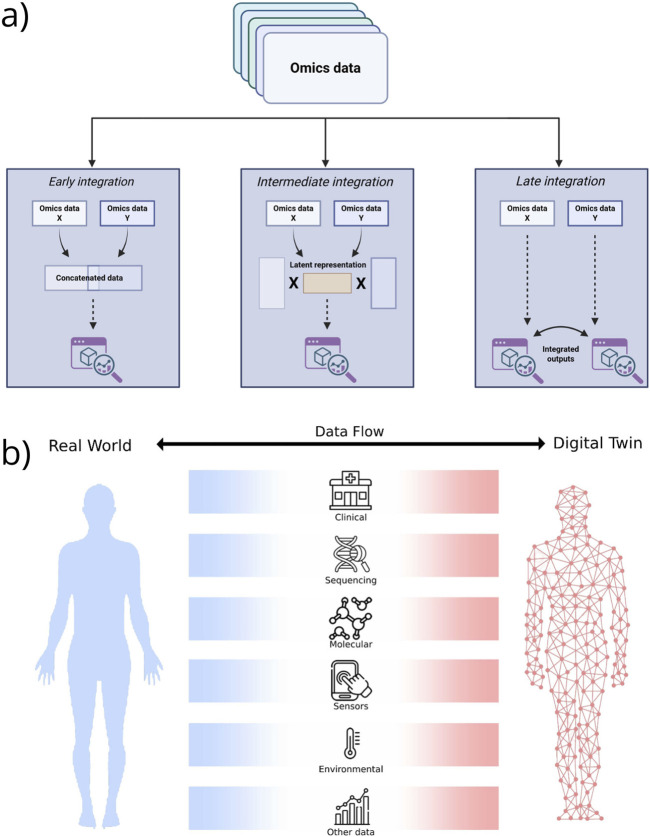
Frameworks for multi-omics integration and DT modeling in cancer research. **(a)** Schematic representation of different strategies for multi-omics integration. **(b)** Conceptual diagram of DTs in the context of cancer research.

Integration of multi-omics data has been an active area of research for more than a decade, leading to several frameworks which can be categorized based on assumptions, computational complexity, interpretability, and the tasks for which they are designed (clinical outcome prediction, mechanistic inference of cross-omics molecular interactions, or dynamic modeling of the temporal evolution of biological systems). Different classes of multi-omics data integration approaches have been proposed, mostly differing in the stage at which data modalities are integrated (Wang et al., 2022; Picard et al., 2021) (Figure 1a).

Early integration techniques typically combine different data types by concatenating features in a single matrix before analysis (Bersanelli et al., 2016). Such an approach is easy to understand and can be easily implemented with many different machine learning (ML) techniques, like regression analysis, clustering or deep learning. This simple approach is often criticized for excessive feature dimensionality and weak interpretability (Hauptmann and Kramer, 2023). Most importantly, it fails to capture the different biological semantics of each data layer and does not explicitly model cross-layer interactions.

Intermediate integration methods, which rely on latent variables or factors, are designed to learn common and/or modality-specific representations across different omics layers. Some examples include matrix factorization, canonical correlation analysis, Bayesian latent variable models and multi-omics factor analysis (MOFA) (Argelaguet et al., 2018). These methods reduce dimensionality while preserving shared sources of variation between datasets, making them particularly useful for patient stratification or biomarker discovery. However, latent variable approaches may trade interpretability for compactness, failing to preserve relationships between molecular entities across different regulatory layers, which is essential for mechanistic models and simulation (Mardinoglu et al., 2025). In DT applications, representations of cross-layer biological interactions and mechanistic detail are essential to simulate system dynamics.

Recent advancements in representation learning further highlight this tension. For example, Joint Embedding Predictive Architectures (JEPA) prioritize the learning of latent representations that are predictive without relying on autoencoder-style reconstruction of the data input (LeCun, 2022), improving robustness but compromising biological interpretability (mathematically useful, not biologically meaningful). Related efforts in single-cell analysis attempt to impose additional structure on latent representations to improve interpretability (Bahrami et al., 2025).

As a result, while techniques of intermediate integration are effective in discovery-oriented and predictive tasks, the abstraction of the latent space makes them less appropriate for building DTs, where interpretability, interaction at different scales, and simulation are critical.

In late integration methods, each omics layer is analyzed independently, typically using modality-specific frameworks, with predictions or learned representations combined only at a later stage, for example, through deep neural networks (Sharifi-Noghabi et al., 2019). This method is advantageous because of its robustness, especially when dealing with structurally different omics layers, but it lacks the ability to capture cross-layer interactions, making it less suitable for DTs.

## Integration strategies across modeling paradigms

Besides the timing of integration, multi-omics approaches can also be classified according to the modeling paradigm that they adopt, namely, linear, deep learning-based and network-based (Table 1).

**TABLE 1:** Taxonomy of integration strategies across modeling paradigms.

Modeling paradigm	Type	Early integration	Intermediate integration	Late integration downstream analysis
Linear methods	Concepts	Feature concatenation PCA on concatenated omics, linear/logistic regression, elastic net, linear SVM	Shared latent factors CCA, joint matrix factorization, multi-block PLS, tensor ICA	Model ensembling Separate regressions per omics, weighted averaging, voting/stacking classifiers
	Tools	None specific	DIABLO (mixOmics); MOFA/MOFA+; RGCCA; Mowgli	None specific
Deep learning-based	Concepts	Input-level fusion Fully connected neural networks, deep autoencoders	Latent-space fusion Multimodal autoencoders, VAEs, contrastive learning models, JEPA-style representations	Prediction-level ensembles Omics stacking, stacked neural networks, DeepEnsemble
	Tools	autoOmics; DeepOmix; flexynesis	MOGONET; MAUI; totalVI; OmiEmbed; OmiVAE; scPairing	MOLI; Super.FELT
Network-based	Concepts	Aggregated network construction Correlation networks from concatenated data	Hybrid latent-network models Multilayer networks, similarity-based fusion, graph-regularized factor models	Separate networks + result integration Independent PPI, GRN, pathway networks with downstream score aggregation
	Tools	None specific	SNF; OmicsIntegrator; netDx	clusterProfiler; EnrichmentMap; WGCNA, ARACNe, GENIE3, CollecTRI, VIPER

Most linear techniques include the early stages of feature concatenation coupled with regression or PCA, intermediate stages that are represented by common latent factor models that employ CCA, MOFA (Argelaguet et al., 2018), DIABLO (Singh et al., 2019) from mixOmics (Rohart et al., 2017) or Mowgli (Huizing et al., 2023), and the late stages that are represented by feature ensembling techniques.

Deep learning-based methods span from early input-level fusion using neural networks to intermediate latent-space fusion via multi-modal autoencoders, variational autoencoders (VAEs), graph neural networks, or contrastive learning frameworks (Ballard et al., 2024), to late prediction-level ensembles like MOLI (Sharifi-Noghabi et al., 2019). These approaches enable cross-modal representation learning but typically operate in abstract latent spaces with limited mechanistic transparency.

In parallel, the field of multi-omics analysis has also been impacted by recent advances in representation learning and foundation models. Specifically focusing on single-cell omics, models such as scVI (Lopez et al., 2018) learn probabilistic latent representations that can be used for alignment, denoising and transfer learning, while the related extension totalVI (Gayoso et al., 2021) extends this framework to multimodal single-cell integration. More generally, predictive representation learning architectures (JEPA) have been proposed to focus on learning abstract latent embeddings that do not require input reconstruction (LeCun, 2022). More recent models, notably scGPT (Cui et al., 2024), have continued this line of research by developing transformer-based models that are pre-trained on large datasets for flexible downstream tasks. Similar foundation-model approaches have also been proposed for biological data, including scPRINT (Kalfon et al., 2025a) and related large-scale biological foundation models (Kalfon et al., 2025b). Although these models are not necessarily developed for multi-omics integration, a trend towards highly abstract latent representations that focus on predictive performance and generalization rather than interpretability is clear throughout the literature.

Approaches based on networks explicitly model relationships, either between samples or between biological entities. Methods such as similarity network fusion (SNF) construct networks of samples for each omics layer and integrate them to improve tasks such as cancer subtyping (Wang et al., 2014). In contrast, multilayer biological network approaches represent interactions between molecular entities across multiple omics layers. Inter-layer edges in such networks can be defined based on different sources of biological evidence, including known molecular relationships (gene-protein mappings), regulatory interactions, or data-driven associations inferred from multi-omics datasets. However, the definition of these edges is often subject to uncertainty, particularly when inferred from noisy or heterogeneous data, which often affect the robustness of the resulting network models. Although these models preserve cross-omics structure and provide a natural framework for mechanistic interpretation, they remain comparatively underexplored in practical multi-omics integration workflows, with only a limited number of recent approaches explicitly implementing such architectures (Trimbour et al., 2024).

Finally, integration strategies also vary based on the structural relationship between the datasets. In vertical integration, multiple modalities are measured on the same samples, while mosaic integration is based on partially overlapping sample sets. Diagonal integration, on the other hand, refers to datasets with non-overlapping samples. These structural differences play a significant role in data integration, especially when integrating heterogeneous datasets (Argelaguet et al., 2020; Cantini et al., 2021). These distinctions are critical for selecting integration methods suitable for DT development.

Beyond multi-omics and multimodal integration, there has been growing interest in the combination of bulk and single-cell transcriptomic data, which is a cross-resolution problem rather than a cross-omics challenge. In this case, the same type of modality is assessed at different biological scales; there is a need for methods that can align signals to reconcile population-level and cell-level variation.

## Implications for digital twins

The development of meaningful biological DTs involves the application of multi-omics integration strategies that extend beyond prediction and specifically capture cross-scale interactions, ensure mechanistic interpretability, and facilitate longitudinal modeling (Mardinoglu et al., 2025). Recent studies highlight the need for DTs to integrate heterogeneous molecular layers with clinical, imaging and phenotypic data in a temporally resolved manner, thus enabling continuous updates of patient-specific models as new data become available (Hsu et al., 2025). Within this setting, emerging multi-omics time series integration frameworks underscore the need to explicitly model longitudinal dynamics rather than relying on the application of cross-sectional latent representations, thus closely aligning with the simulation and prediction objectives of DTs (Sherwani et al., 2025).

In addition, a number of recent conceptual models further suggest that DTs should, in fact, be computational avatars that integrate various omics-based variables with clinical variables, facilitating identification of new biomarkers that can be used for early detection, improved tumor diagnosis and therapy optimization (Lococo et al., 2023). Digital Human Avatars models stress the importance of a structured model, such as hybrid mechanistic/statistical models or networks, that is biologically meaningful but also computationally tractable. In a complementary direction, a number of recent perspectives in cancer systems biology stress that, in order to be biologically plausible, robust and trustworthy, DTs should integrate data-driven learning with mechanistic constraints (Asghar and Chung, 2025).

Taken together, these perspectives indicate that integration methods optimized for predictive performance alone, such as latent variable or black-box deep learning models, are not sufficient for DT implementations, which require mechanistic and longitudinal approaches to simulate interventions (Mardinoglu et al., 2025). Network models and hybrid models, especially in their time-evolving and patient-specific variants, provide a crucial methodological foundation for the development of the next-generation of clinically actionable DTs.

## Digital twins in precision health

### Definition and concept

By offering opportunities for individualized healthcare through virtual models, DTs represent a paradigm shift in the field of precision health and personalized medicine. In healthcare, DTs can be defined as highly detailed and dynamic virtual replicas of physical entities (cells, tissues, organs, patients and health systems), reproducing the structure, behavior and context of their physical counterpart (Qi et al., 2021). These virtual replicas are continuously updated with real-time data to simulate and predict health outcomes, thereby optimizing clinical decision-making (Fuller et al., 2020). DTs integrate data from multiple sources in real-time to simulate health outcomes and can be adapted to each patient, potentially increasing proactive healthcare management by enabling more precise, timely and effective clinical interventions, ultimately improving patient treatment efficiency.

Beyond computational models, DTs are not limited to simple static digital models; they involve a bidirectional flow of information between the physical entity and its digital counterpart, ensuring that the virtual model evolves at the same time as its real-world counterpart (Figure 1b). This continuous synchronization enables DTs to reflect the actual current state of the physical entity accurately, as well as predict future states under various scenarios (Kamel Boulos and Zhang, 2021). For instance, in a clinical setting, DTs of a patient would integrate data from various sources (clinical records, genetic data, lifestyle information, real-time monitoring from wearable devices, etc.) to provide a comprehensive dynamic representation of the patient’s health status and guide recommendations for therapeutic interventions or increased surveillance.

The effectiveness of DTs depends on their predictive capability (Fuller et al., 2020). Leveraging real-time data and historical health information helps DTs forecast various factors such as disease progression, outcomes of different treatment options, or early signs of health deterioration. This predictive capability is particularly valuable in the management of chronic diseases, where early intervention can significantly influence disease progression. DTs can be used not only for optimizing disease treatments, but also to suggest strategies for preventing disease onset and mitigating its impact. Their predictive power comes from modeling disease trajectories, i.e., mapping a disease’s progression over time, including its onset, development and chronicity, when relevant, starting from clinical records, multi-omics data and real-time health metrics. Similar data-driven approaches have also been applied to predict disease risk and evolution using longitudinal health data and ML (Lasko et al., 2025). Therefore, they offer the potential for medical professionals to proactively adjust treatment plans, and anticipate significant turning points in the disease’s course. They can also be used to provide alerts for early indicators of health deterioration for chronic diseases, whose trajectory frequently includes periods of remission or relapse.

Moreover, the DTs framework can help in the creation of a learning healthcare system where continuous feedback from DTs informs clinical decisions and enhances healthcare delivery. This iterative process not only improves individual patient care, but also contributes to the broader knowledge base, driving key advancements in medical research and clinical practice (Kamel Boulos and Zhang, 2021).

### Potential and existing applications of DTs or DT-like models in healthcare

There are three main uses that DTs could have in healthcare, namely, disease management and treatment optimization, virtual clinical trials, and patient education.

Personalized medicine and disease management are a few of the areas in which DTs are gaining recognition for their transformative potential in the healthcare industry. Several existing systems integrate multi-omics data with clinical and lifestyle information to create a more complete and individualized health profile that can serve as a basis for *in silico* disease modeling. These models can form the basis for clinical decision support systems, providing predictions of responses to the different treatment options, a highly valuable tool for clinicians who can tailor interventions based on the unique characteristics of each patient. In addition, DTs enable customized interventions by utilizing real-time data to offer precise and dynamic insights into patient health; this involves continuous mapping of the physical/digital counterparts.

Virtual representations play an important role in the prevention and management of chronic disease. The constant monitoring of physiological data allows DTs to identify early signs of disease onset but also aggravation, which is crucial for timely intervention.

In the context of diabetes management, DTs have demonstrated the capacity to predict glucose fluctuations by integrating data on dietary intake, physical activity or medication adherence, making them a powerful tool to help clinicians with better management of the disease and preventing complications (Shamanna et al., 2021). Recently, this has led to the design of DT systems that administer insulin through real-time monitoring of patient glucose levels (Thamotharan et al., 2023). Other studies in cardiovascular health demonstrate how DTs can monitor vital or relevant cardiovascular signs (resting heart rate, heart rate variability, blood pressure) to detect abnormalities at an early stage and suggest preventive measures (Coorey et al., 2022).

Specifically in oncology, advanced computational frameworks have long been used for modeling tumor growth and predicting how a tumor will respond to different therapeutic interventions (Benzekry et al., 2014), leading to optimization of cancer patient treatment plans by improving drug effectiveness and minimizing side effects (Bruynseels, Santoni de Sio and van den Hoven, 2018). Recent work at the interface of mathematical oncology and AI further emphasizes the role of computational models in survival prediction (Rockne et al., 2026; Taieb et al., 2026).

However, only a few real DTs for oncology have been developed so far. This is due to both the difficulty in obtaining longitudinal data, and the near-impossibility of dynamically adapting treatments in response to model predictions. As a result, the systems currently described in the literature often lack bidirectional feedback between the real system and the digital counterpart, suggesting that what we discuss here could be referred to as Digital Shadows, as discussed in (Pellegrino et al., 2025).

Some studies demonstrated a significant increase in treatment efficacy that shows the potential of mathematical models that are similar to DTs in adapting cancer therapies. Simulations predicting a trajectory for a patient’s cancer under one of the treatment options can be used to adapt treatment (Hernandez-Boussard et al., 2021). Additional examples of oncological DTs are reviewed in (Stahlberg et al., 2022), illustrating the limits but also the potential of DTs to improve early diagnosis of cancers that are generally detected at advanced stages. Some projects on the detection of pancreatic cancer found subtle changes in biomarker profiles that could be detected at an early stage, potentially indicative of the initial stages of pancreatic cancer (Boyd et al., 2023). However, matching real patients in cohorts with simulated patients from the model did not result in a definitive mapping between real-world data and model parameters (Junet et al., 2023). Another project involved using simulations of cellular interactions in the tumor to personalize melanoma treatment, showing that computational models integrating tumor growth or immune response could predict patient-specific outcomes in melanoma therapy (Perlstein et al., 2019; Hodgkinson et al., 2022).

Another remarkable application predicts drug efficacy in lung cancer using DTs (Wang et al., 2024). DT models allow the simulation of different delivery methods and dosages under different therapeutic scenarios to establish treatment efficacy. Understanding these mechanisms is essential for clinicians to be able to adjust treatment plans as a therapeutic or preventive measure (Vallée, 2024). Some studies also underscored DTs’ value in the anticipation of treatment response in cancer therapy (Venkatesh et al., 2024).

On the other side, further from real patients, testing new drugs or treatments via virtual platforms is an application of DTs that is transforming drug discovery. The simulation of biological processes and disease progression is implemented in DTs to predict the interaction of new compounds with different molecular targets, observing how these processes or the disease will evolve in simulated diverse patient populations constituting virtual cohorts (Wang et al., 2024). This approach reduces the need for extensive *in vivo* testing, improving the drug development process.

Finally, DTs could also play a major role in patient education, by improving their own awareness and encouraging them to be proactive about their health. If an interactive representation of health status via Patient Reported Outcomes and the impact of different lifestyle choices is provided to patients, DTs could indirectly enhance motivation for self-management. For instance, DTs can show some patients with obesity the impact of weight loss on some health metrics, encouraging changes in diet and exercise regimens (Gkouskou et al., 2020).

These examples show that the applications of DTs in healthcare are broad: personalized medicine, disease prevention/management, drug discovery and patient engagement. The integration of real-time data by DTs offers dynamic insights that ameliorate personalized healthcare. As the technology continues to evolve, the potential of these DTs to improve healthcare outcomes should continue to grow, highlighting their importance in the future of precision health (Kamel Boulos and Zhang, 2021). Examples from the literature reveal the potential of dynamic and predictive models to improve early detection, personalize treatment strategies, or anticipate therapeutic resistance. However, the development of tools that enable feedback between the DT and real patients is still in progress (Fuller et al., 2020), holding promise in cancer research.

### Integrating multi-omics data in DTs of cancer patients: challenges and opportunities

A full comprehension of molecular mechanisms driving cancer progression is made possible by the integration of multi-omics data, which has become an essential component in oncology. The word “omics” refers to a group of scientific areas, each of which focuses on specific biological entities (Srivastava et al., 2024). Genomics, epigenomics, transcriptomics, proteomics, metabolomics, metagenomics and even radiomics (medical imaging such as MRI, CT scans, etc.) are the main categories of data that are important for cancer research. We give an overview of different types of omics data, their general characteristics and relevance to cancer research in Table 2.

**TABLE 2:** Different types of omics data, their characteristics and relevance to cancer research.

Omics type	Description	Technologies	Applications to cancer research
Genomics	Study of DNA sequence, identifying mutations and genetic alterations driving disease	Whole-genome sequencing (WGS), Whole-exome sequencing (WES), liquid biopsy (ctDNA sequencing)	Identification of mutations in oncogenes, tumor suppressor genes, and genetic alterations, non-invasive tumor profiling and monitoring through circulating tumor DNA
Epigenomics	Study of changes in chromatin that do not involve changes in the DNA sequence, including DNA methylation, histone modifications, chromatin accessibility and organization	ChIP-seq, Cut&Run, ATAC-seq, Hi-C	Understanding epigenetic regulation and gene silencing
Transcriptomics	Study of RNA transcripts, providing insights into gene expression	RNA sequencing (RNA-seq), single-cell RNA-seq (scRNA-seq)	Identification of dysregulated genes, tumor heterogeneity, molecular subtypes, and patient stratification
Proteomics	Large-scale study of proteins, including their expression, modifications, and interactions	Mass spectrometry (LC-MS/MS), protein arrays	Identification of altered protein expression, protein biomarkers, and targets for therapy in cancer
Metabolomics	Study of metabolites, small molecules that are intermediate products of metabolism, offering a functional readout of biochemical activities	Nuclear magnetic resonance (NMR), Mass spectrometry (MS)	Characterization of metabolic alterations in cancer, such as the Warburg effect, and understanding of their role in tumor development
Metagenomics	Analysis of the genetic material from microbial communities, in the gut/saliva/skin or at tumor sites, offering insights into the microbiome’s role in cancer	Shotgun metagenomic sequencing, 16S rRNA sequencing	Exploration of microbiome influence on tumorigenesis and potential microbiota-based cancer therapies
Spatial omics	Study of the spatial organization of cellular components within the TME, preserving tissue architecture during analysis	Spatial transcriptomics, spatial proteomics	Visualization of cellular heterogeneity and spatial patterns in tumors
Exposomics	Study of lifetime environmental exposures and their interaction with the genome, focusing on external and internal factors such as chemicals, diet, and lifestyle	Data mostly from environmental sensors, wearable devices, or questionnaires	Understanding of environmental factors contributions to cancer risk through gene-environment interactions

The integration of multi-omics data in oncology offers significant potential to advance the understanding of cancer biology. However, several challenges must be addressed to fully realize its promise. These challenges can be technical, computational and biological in nature, also including issues with data administration policies, each representing unique opportunities for innovation.

One of the main issues in multi-omics integration is the inherent heterogeneity of the data, stemming from both its nature and its method of generation. Different omics data sources are generated using diverse technologies, each having its own data format (Cao et al., 2021; Ren et al., 2023), indicating the necessity for a deeper investigation of the crosstalk between different levels of data granularity (Cirillo et al., 2021). This inconsistency adds complexity to the integration process, because the harmonization and standardization of these datasets require advanced methodologies (Hasin et al., 2017).

The high volume of multi-omics data requires efficient algorithms for extracting useful and interpretable information. Also, this high-dimensional data integration involves handling large-scale datasets that demand high computational power as well as scalable storage capacity (Nikolski et al., 2024). Moreover, the development of efficient algorithms for the different steps of data processing (preprocessing, normalization and integration) is crucial to deal with this complexity (Santiago-Rodriguez and Hollister, 2021). ML or AI techniques are increasingly employed to address these challenges (Rockne et al., 2026), but their implementation requires substantial expertise and resources (Angermueller et al., 2016; Libbrecht and Noble, 2015; Cai et al., 2022; Acharya and Mukhopadhyay, 2024).

Ensuring data quality and reproducibility is another significant challenge. Omics data can be affected by various technical issues at different steps of the experiments or analysis pipeline (technical biases, batch effects, sample-specific variations, etc.), which can hide biological signals and lead to false conclusions (Leek et al., 2010). Therefore, rigorous quality control measures, standardized protocols and reproducible workflows are essential to mitigate all these issues. Additionally, the integration process must account for missing data and varying sample sizes across different omics layers (Kim et al., 2005).

On another level, complex datasets are generated by the integration of multi-omics data, which requires careful interpretation to derive biologically meaningful insights. For instance, the identification of key drivers in cancer and the elucidation of their functional roles in the context of multi-omics data is an important and challenging task. Distinguishing true biological signals from noise and correlating multi-omics findings with clinical outcomes is non-trivial. Collaborative efforts between researchers and clinicians are essential to contextualize the results and then translate them into actionable knowledge (Demirel et al., 2022).

**FIGURE 2:**
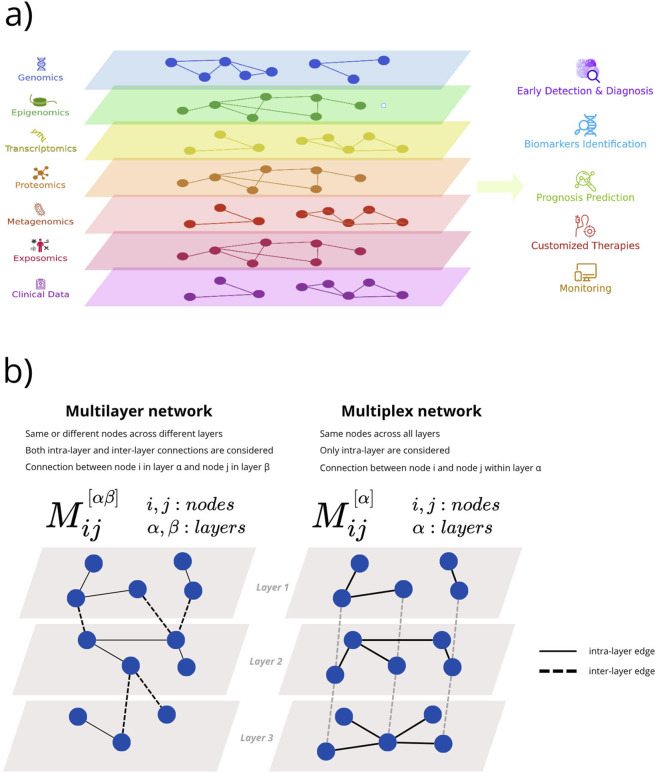
Multi-omics interactions and their network-based representation in therapeutic modeling. **(a)** An overview of different multi-omics layers and their potential applications in clinically relevant tasks. **(b)** Schematic representation of multilayer and multiplex networks. A multilayer network showing intra- and inter-layer connections (left) and a special case of a multilayer, a multiplex network, in which the nodes are the same in all layers (right).

Despite all these challenges, the integration of multi-omics data currently offers substantial opportunities to deepen our understanding of cancer biology at the tumor site and across the whole organism. The combination of information from several types of omics data can bring an extensive view of the molecular mechanisms that drive tumor growth via different types of data integration (Huang et al., 2017) (Figure 2a). This global approach helps to identify new biomarkers that may not be apparent from single-omics studies alone, with some methods taking into consideration data type diversity and big data volume (Wu et al., 2015).

Great progress can be achieved in personalized medicine thanks to multi-omics integration. The detailed molecular profiling of individual tumors can reveal patient-specific vulnerabilities, to enable designing customized therapeutic strategies for the patients. For instance, integrating genomic and transcriptomic data is interesting to identify actionable mutations or dysregulated pathways, while transcriptomic, proteomic and metabolomic analyses can improve the understanding of tumor metabolism alterations and cellular phenotypes (Zhang and Plevritis, 2018; Gegner et al., 2023).

Developing novel algorithms or ML models to handle high-dimensional, heterogeneous data is another emergent area of research (Wu et al., 2019). These advancements not only improve the performance (efficiency and accuracy) of data integration, but also contribute in a broader way to the field of computational biology. The complexity of the integration makes collaborative efforts and data sharing among researchers and institutions absolutely necessary (Gómez-López et al., 2019). Some initiatives, such as The Cancer Genome Atlas (TCGA) (Weinstein et al., 2013), International Cancer Genome Consortium (ICGC) (Zhang et al., 2011) and Human Cell Atlas (HCA) (Rozenblatt-Rosen et al., 2017), provide highly valuable resources and infrastructure for multi-omics research. Some recent resources are specific to certain types of omics data, such as the Human Protein Atlas (Pontén et al., 2008) (proteomics), International Human Epigenome Consortium (IHEC) (Stunnenberg et al., 2016) (epigenomics), Human Metabolome Database (HMDB) (Wishart et al., 2007) (metabolomics), or the Virtual Metabolic Human (VMH) database (Noronha et al., 2019), which provides curated metabolic knowledge and genome-scale models linking metabolism and microbiome interactions. These collaborative platforms are excellent for the exchange of data, tools or expertise, accelerating scientific discoveries and clinical translation.

## Multilayer network approaches for multi-omics integration

### Fundamentals of multilayer networks

Multilayer networks are a powerful framework to model complex systems characterized by multiple types of interactions (or relationships). Unlike traditional single-layer networks, which represent a single type of interaction among molecular entities, multilayer networks incorporate multiple interconnected layers, where each of them represents a different type of relationship, capturing the multifaceted nature of real-world interactions (Kivela et al., 2014; De Domenico, 2022). More specifically, a multilayer network is formally defined by a set of nodes and layers, where each node can exist in one or more layers, while edges represent the connections between nodes within the same layer (intra-layer edges) or across different layers (inter-layer edges) (Figure 2b). The interconnections between the different layers (inter-layer edges) are key elements in the network to understand the influence of different types of interactions and the impact on the overall dynamics of the system.

One of the main advantages of multilayer networks, compared to single-layer networks, comes from their ability to model interdependencies between different types of data. In systems biology, a multilayer network can simultaneously represent genomic, transcriptomic, proteomic and clinical data, each as a distinct layer. The interactions within each layer capture relationships specific to that data type, such as transcription factors (TFs) - target genes (TGs) regulatory interactions in the transcriptomic layer or protein-protein interactions (PPI) in the proteomic layer. On the other hand, inter-layer edges capture the relationships between different data types. The construction of multilayer networks involves several methodological steps:

1. Data preprocessing to ensure compatibility: Ensures consistency across omics datasets, involving quality control, noise reduction, and standardization. Batch effect removal and data imputation address technical biases and missing values to accurately reflect biological variability (Leek et al., 2010; Lazar et al., 2013).
2. Single-layer network reconstruction: For each omics type, individual single-layer networks are constructed, based on annotated interactions such as TF-target interactions, PPI, or metabolic pathways. Algorithms tailored to each data type can establish connections indicating correlation, causality, or interaction strength, with edge direction or weight based on interaction type and confidence.
3. Integration into the multilayer network: Single-layer networks are integrated into a multilayer network via inter-layer edges, representing cross-‘omics interactions, i.e., regulatory relationships between genes and proteins, or other molecular entities. Several approaches can be used to define inter-layer edges, including data-driven methods that infer relationships based on statistical correlations or ML models that predict interactions based on prior knowledge of biological principles. A special case of multilayer networks is obtained when all nodes are present in all layers, denoted as multiplex networks, where distinct layers represent different interaction types for the same set of nodes (Figure 2b), preserving the individual network topologies (Kivela et al., 2014; Valdeolivas et al., 2019).

The constructed network can be visualized using *ad hoc* developed tools, or extensions of other tools initially built for single-layer network visualization, which we summarize in Table 3.

**TABLE 3:** Tools used to reconstruct, visualize and analyze multilayer networks.

Tools	Description
MUXViz (De Domenico et al., 2015b)	Visualization of multiplex networks, allowing the exploration of multiple layers of interactions in the same system
Omics Integrator (Tuncbag et al., 2016)	Incorporation of different omics data into coherent networks, optimized for gene, protein and pathway interactions
NetZoo (Ben Guebila et al., 2023)	Collection of methods for inference and analysis of gene regulatory and multi-omic networks (like PANDA, LIONESS, DRAGON), for construction and downstream analysis of complex multilayer biological networks
MultiXrank (Baptista et al., 2022)	Random walk with restart on heterogeneous multilayer networks, used for node prioritization and exploration of information flow across layers
Cytoscape (Shannon et al., 2003); NetworkX (Hagberg et al., 2008); iGraph (Csárdi et al., 2006); Gephi (Bastian et al., 2009)	General-purpose network visualization tools, can be adapted for multilayer network exploration and custom multilayer implementations. Often used for custom multilayer implementations

#### Analysis of multilayer networks

The advancement of computational tools, algorithms and graph-based methods is essential to analyze multilayer networks and interpret their features. One approach is the use of centrality measures to determine the importance of nodes within the multilayer network. Frequently used centrality measures for single-layer networks (degree, betweenness centrality, alpha centrality, etc.) are extended to consider the multilayer context, offering the opportunity to find nodes that play a major role across multiple layers, or, even further, to identify functional modules (De Domenico and Lancichinetti, 2015). Another essential technique is community detection, an analytical technique which involves node clustering via community identification, by identifying sets of nodes that are densely connected amongst each other within and across layers compared to the connections they have with other nodes not in the community. This approach reveals functional modules of nodes that work together in a coordinated way in several contexts (Mucha et al., 2010; Didier et al., 2015), particularly in complex diseases (Choobdar et al., 2019; Halu et al., 2019).

Additionally, one can examine multilayer motifs: small recurring patterns of interconnections that occur within and between network layers. These motifs establish the fundamental building blocks of the network’s structure (Boccaletti et al., 2014). More advanced concepts, such as the Von Neumann entropy of a multilayer network, can be utilized to characterize global connectivity patterns across layers (De Domenico et al., 2015a).

Studying network dynamics is also relevant to understand how information propagates through the multilayer network (a phenomenon called flow) via modeling the spread of perturbations and to investigate how interactions in one layer affect dynamics in other ones (Gómez et al., 2013; De Domenico et al., 2016). One of the most used approaches for this purpose is Random Walk with Restart, which identifies relationships between nodes, reconstructs communities and predicts key interactions (Cowen et al., 2017; Baptista et al., 2024).

#### Machine learning and predictive modeling

ML techniques are increasingly applied to multilayer networks to develop predictive models. Supervised learning algorithms, like support vector machines (SVM) or random forests (RF), can be trained on multilayer network features to predict clinical outcomes or response to therapy. Unsupervised learning methods (clustering or dimensionality reduction) can help to discover new patterns or possibly some associations within the integrated data (Wu et al., 2019). Predictive modeling using multilayer networks enhances the ability to make data-driven decisions in personalized cancer treatment.

Network embedding approaches are also effective in analyzing a wide range of networks, even if they were not originally developed for biological networks (Parvizi et al., 2022). These methods have shown good performance in tasks like community detection, node classification and link prediction. However, most embedding methods are not designed to be applied to multiplex networks. To address this, advanced methods like MultiVERSE were developed and shown to have superior performance in tasks such as link prediction and network reconstruction, particularly in multiplex networks (Pio-Lopez et al., 2021).

With their ability to learn complex relationships and inherent structures in multilayer networks, Graph Neural Networks (GNNs) are highly effective at analyzing graph-structured data (Corso et al., 2024). They encode both the node features and the graph topology into low-dimensional embeddings, which can then be used for a variety of tasks, such as node classification and link prediction (Zhang, 2022). Latest advancements, like Graph Convolutional Networks (GCNs) and Graph Attention Networks (GATs) enhance the capacity to represent heterogeneous data and facilitate tasks such as drug target prediction (Grassia et al., 2021; Chatzianastasis et al., 2023; Chen et al., 2024).

#### Applications of multilayer networks in data integration

Data integration based on multilayer networks is performed by the inference or construction of integrated networks that combine multi-omics data. This all-around approach is ideal to map the interactions between different molecular entities across several biological layers, leading to a multidimensional view of cellular processes. Genomic data can reveal mutations in specific genes, while transcriptomic data can show how these mutations affect gene expression levels. Integrating these datasets into a multilayer network using approaches like HuMMuS (Trimbour et al., 2024) enables the reconstruction of molecular regulatory mechanisms from single-cell multi-omics data by capturing both intra-omics (peak-peak, gene-gene, TF-TF) and inter-omics (peak-gene, TF-peak) interactions; this could be a way to establish the influence of genetic alterations on transcriptional changes and possibly the propagation of these changes through proteomic and metabolomic layers.

A few diverse multilayer networks have been constructed, for instance integrating disease-perturbed proteins, drug targets and biological functions into a multi-level interactome network for a better definition of disease mechanisms (Ruiz et al., 2021), while similar approaches employing multilayers have also been applied to the study of specific pathologies, such as infection by SARS-CoV-2 (Messina et al., 2020; Verstraete et al., 2020).

To conclude, in cancer research, significant benefits emerge from the application of multilayer network theory, especially the integration of diverse types of biological data that enhances the view of the molecular mechanisms driving tumors. This integrative approach can help to identify key regulatory pathways and potential therapeutic targets that might be overlooked in single-layer analyses. Overall, multilayer networks can help to interpret the complex interplay between genetics and environment (epigenetic modifications, etc.) in cancer evolution.

#### Digital twins for cancer research: a multilayer network perspective

The conceptual framework for utilizing DTs in oncology through a multilayer network perspective covers a systematic approach that integrates diverse types of biological data to create predictive models of tumors. This framework leverages the positive aspects of multilayer network theory to deliver a more complete representation of the biological processes and molecular functions involved in cancer progression (Laubenbacher et al., 2022). In the following sections, we consider the multilayer network as the scaffold of a patient DT and give a roadmap of how to build it by integrating multi-omics data, as well as how to use it in cancer research.

The integration of multi-omics data is the core of this conceptual framework, combining diverse molecular layers, and eventually complementary patient-level information such as clinical records and host-derived data (blood readouts as in liquid biopsies, metagenomics of the microbiome, exposomics, etc.). Each of these layers brings unique and complementary insights into the different aspects of cancer.

The final step in the framework corresponds to the clinical implementation and validation of the DT, a challenging but major task for the technology to gain credibility. In this context, two different concepts exist. The first one corresponds to the integration of DTs into clinical workflows, where they can assist oncologists in the decision-making process with data-driven choices regarding diagnosis, prognosis or treatment (clinical decision support systems). The second one is based on the use of DTs to perform virtual clinical trials, testing the efficacy of new compounds or even drug associations in a virtual environment before applying them to patients to limit the risk (Wang et al., 2024). However, the validation of DTs remains a significant challenge. Retrospective validation can be done using held-out patient cohorts, whereas rigorous strategies in cross-validation, including nested cross-validation, should be used to reduce the risk of overfitting in a high-dimensional multi-omics environment. External validation across independent cohorts is essential in the overall process. Furthermore, the methodology is challenged by the potential issue of circularity in using the same omics data in network construction and downstream predictive modeling. Validation of DTs’ predictive capabilities is indispensable to ascertain their reliability. Some retrospective analysis using historical patient data, as well as prospective validation in clinical trials like treatment timing optimization can be performed. Continuous monitoring and updating of DTs with dynamic, up-to-date patient data would reinforce the accuracy and confirm the effectiveness of these tools for cancer management (Hernandez-Boussard et al., 2021).

### Practical challenges in DT implementation

#### Research gaps

The scarce availability of longitudinal data to capture the dynamic nature of tumor progression is a significant challenge. This causes a limited understanding of temporal dynamics in cancer progression or even treatment response. Continuous monitoring and data acquisition in real-time from patients involves the integration of clinical, imaging, liquid biopsy or wearable device data, as well as potentially patient-reported outcomes, with multi-omics profiles (Chow et al., 2024). Achieving this level of integration requires advanced data management systems that are capable of handling diverse data flows, while also maintaining temporal consistency, such as those employed in personalized nutrition applications (Romero-Tapiador et al., 2026).

A very important issue that should not be neglected is the use of sensitive personal health information because it raises important ethical and legal concerns related to privacy, consent, and data ownership. Among them, protecting patient data from unauthorized access is critical as data breaches can lead to potentially severe consequences, like identity theft or loss of trust in healthcare systems (Rothstein, 2015; Thapa and Camtepe, 2021). Data privacy should be made a priority by implementing stringent data governance policies to protect patient information. Techniques such as differential privacy, which add noise to datasets to prevent the identification of individuals, are increasingly employed to protect patient confidentiality while allowing data analysis (Dwork and Roth, 2014). Recently, promising solutions have been suggested, such as blockchain, which offers a decentralized and immutable ledger that can be used to securely store/share patient data (Pournaghi et al., 2020), or homomorphic encryption, allowing computations on encrypted data without needing to decrypt it first (Munjal and Bhatia, 2023). Security measures must address the risk of cyber-attacks with the implementation of robust cybersecurity protocols (intrusion detection systems, secure access controls, etc.), which is essential to protect multi-omics data and DT models from hostile attacks, and must also comply with evolving regulatory frameworks governing the use of health data. In Europe, the General Data Protection Regulation (GDPR) and the EU AI Act impose requirements on data privacy, transparency and accountability, particularly for high-risk AI systems in healthcare. In the US, the Health Insurance Portability and Accountability Act (HIPAA) governs the use of protected health information, while guidance from the Food and Drug Administration (FDA) applies to AI/ML-based software as a medical device. These regulatory considerations can represent important challenges for the clinical deployment of DTs.

The biological interpretation of integrated multi-omics data is another research gap. While multilayer networks can reveal complex interactions, translating these findings into concrete biological insights remains a challenge. There is a need for new tools to interpret the results of multilayer network analyses in a technically and biologically meaningful way. In addition, experimental validation of the predictions made by DTs is necessary to confirm different parameters, like accuracy or utility in clinical settings.

### Data collection and AI

The adoption of DTs in precision medicine is increasingly operational, driven by extensive data collection alongside traditional biomedical methodologies. However, the reliance on black-box predictive models based on large datasets presents limitations that could potentially slow down the broader application of DTs in clinical settings. In the literature, it has been argued that hypothesis-driven generative models, which generate data based on a set of underlying assumptions of the processes that generate the observed data, more particularly multi-scale modeling, are essential to boost the clinical accuracy of DTs. The transformative potential of DTs in healthcare has been explored by emphasizing their capability to simulate complex interdependent biological processes across multiple scales. The integration of generative models with extensive datasets can deliver scenario-based approaches to explore diverse therapeutic strategies. It is an excellent strategy to support dynamic clinical decision-making. This method not only leverages advancements in data science to improve disease management, but it also incorporates insights from complex systems, quantitative biology and digital medicine to improve patient care (De Domenico et al., 2025).

### Perspectives

In the context of cancer, multilayer networks have been used to capture complex interactions, leading to the identification of novel therapeutic targets (Liu et al., 2020). The integration of gene expression data with chromatin accessibility and protein-protein interaction networks was shown to uncover key regulatory proteins that are central to tumor progression, which might not be clearly identified when examining single-layer networks, highlighting the added value of a multilayer approach to propose new biomarkers.

Given the importance of the tumor microenvironment (TME) in affecting tumor progression and therapy response (Quail and Joyce, 2013), it is imperative to explore the integration of information regarding all cellular populations within a tumor sample into predictive models. The TME refers to the complex ecosystem surrounding a tumor composed of immune cells, fibroblasts, endothelial cells, extracellular matrix, blood vessels, and signaling molecules, all of which influence tumor growth. This process requires the non-trivial development of new methods that capture salient information from either estimation of cell type proportions from bulk RNA-seq (Merotto et al., 2024), or from single-cell spatial (Visium/Xenium) (Ståhl et al., 2016) and non-spatial datasets (10x Genomics scRNA-seq/scATAC-seq) (Zheng et al., 2017). It is likely that new modes of dimensionality reduction will have to be applied to these types of data to produce correspondences between these single-cell level descriptions and more traditional layers, in which nodes are molecular entities. In this context, node identity shifts from molecular entities to cells or cell types, and multilayer networks can incorporate additional layers capturing cell-cell communication using methods such as NicheNet, CellChat, LIANA (Sang-aram et al., 2025; Jin et al., 2025; Dimitrov et al., 2024). Recent tools for gene regulatory inference (SCENIC+, ArchR) (Bravo González-Blas et al., 2023; Granja et al., 2021), multimodal integration (Seurat WNN), and spatial analysis (Squidpy) (Palla et al., 2022) further illustrate these developments.

Multi-level and hybrid models will become necessary to use the multilayer structures beyond simple data integration and towards dynamic modeling (Pancaldi, 2026). Despite the obvious interest in analyzing the integrated datasets on this multilayer structure, looking for communities spanning several layers, for example, the real power of this framework will come from the construction of dynamic models of these complex systems. A first approximation of dynamics can be provided by flow analyses on the network, which simulate how the information will spread within and across layers, providing an idea of perturbations that can move the DT towards a target state (for instance, towards a healthy state). Simulating the dynamic state of a patient, including the tumor itself, the immune system, different organs and how the patient environment is impacting all of them will probably require a combination of models of different scales (e.g., molecular, metabolic, cellular, etc.) as well as a better understanding of regulation and correspondence between nodes in different layers. Further down the line, with a better understanding of the underlying multi-level biological processes, we might be able to construct executable models comprising the entire variety of datasets, constituting DTs that will be extremely useful for *in silico* clinical trials that consider the patient as a whole (Clarke and Fisher, 2020; Niarakis et al., 2024).

Several approaches to modeling the TME have been proposed (Metzcar et al., 2019; Johnson et al., 2025), and these might enable a dynamic perspective, which is essential to understand the adaptation of tumors to therapeutic molecules. More effective treatment strategies might require combinations of different approaches at different times, requiring a good understanding of the time scales of the different biological processes involved in cancer (Wang and Deisboeck, 2019).

To move closer to virtual clinical trials, a more complete representation of patients would be required. The flexibility of the multilayer approach should enable the integration of diverse data types from the tumor and from the patient as a whole. For instance, integrating prior comorbidities could be of great value in predicting patient outcomes, as was the case for predicting pancreatic cancer onset (Placido et al., 2023). Indeed, patient comorbidity profiles might work as proxies for identifying patient endotypes or characteristics that are relevant for patient stratification (Sánchez-Valle et al., 2020), while integrating exposures or side effects with tumor characteristics via multilayer networks and multi-level models could produce more useful DTs for drug discovery and development of *in silico* clinical trials.

## Conclusion

This review has examined the methodologies, tools, and challenges in the implementation of multilayer networks as cornerstones of DTs for cancer, building on their potential to provide a comprehensive and integrated view of the patient, a central element required to predict disease progression, response to treatment, and side effects. Despite the hopes in DTs, some challenges remain, specifically in data integration, computational efficiency, and data privacy. Future research should focus on developing computational models and enhancing scalability, without neglecting ethical frameworks for patient data use. Collaboration between the private sector, clinicians and researchers could help overcome these challenges. DTs offer opportunities to transform cancer care with more effective and individualized treatments by providing dynamic, multiscale models that simulate tumor biology and patient trajectories over time. Further advancements in this field, with the integration of more patient-level data, are crucial, enabling better *in silico* clinical trials and accelerating cancer treatment development.

## Glossary

Multi-omics integration: The process of combining data from multiple omics layers to obtain a more comprehensive understanding of biological systems
Multilayer network: A network structure in which multiple relationship types or data modalities are represented as distinct but connected layers, allowing the modeling of complex biological systems across different scales
Multiplex network: A specific class of multilayer networks in which the same set of nodes is connected across layers through different types of interactions
Inter-layer edge: A connection linking nodes across different layers of a multilayer network, representing relationships between entities at distinct biological levels
Digital Twin (DT): A dynamic, patient-specific computational model that integrates multi-scale data to simulate biological processes, disease progression, or potential responses to therapeutic interventions
Tumor microenvironment (TME): The complex cellular and molecular environment surrounding tumor cells, including non-cancerous cells, such as immune cells and stromal cells, signaling molecules, and extracellular matrix components Intra-tumor
Intra-tumoral heterogeneity: The presence of diverse cellular, genetic and molecular profiles within a single tumor
